# In Vitro and Predictive Computational Toxicology Methods for the Neurotoxic Pesticide Amitraz and Its Metabolites

**DOI:** 10.3390/brainsci13020252

**Published:** 2023-02-01

**Authors:** Marialuce Giorgini, Mercedes Taroncher, María-José Ruiz, Yelko Rodríguez-Carrasco, Josefa Tolosa

**Affiliations:** Laboratory of Food Chemistry and Toxicology, Faculty of Pharmacy, University of Valencia, Av. Vicent Andrés Estellés s/n, 46100 Burjassot, Spain

**Keywords:** neurotoxicity, cytotoxicity, in silico methods, amitraz, metabolites

## Abstract

The *Varroa destructor* parasite is responsible for varroasis in honeybees worldwide, the most destructive disease among parasitic diseases. Thus, different insecticides/acaricides have been widely used within beehives to control these parasitic diseases. Namely, amitraz is the most used acaricide due to its high efficacy shown against *Varroa destructor*. However, pesticides used for beehive treatments could be incorporated into the honey and accumulate in other hive products. Hence, honeybee health and the impairment of the quality of honey caused by pesticides have gained more attention. Amitraz and its main metabolites, N-(2,4-dimethylphenyl) formamide (2,4-DMF) and 2,4-dimethylaniline (2,4-DMA), are known to be potent neurotoxicants. In this research, the cytotoxicity of amitraz and its metabolites has been assessed by MTT and PC assays in HepG2 cells. In addition, possible target receptors by in silico strategies have been surveyed. Results showed that amitraz was more cytotoxic than its metabolites. According to the in silico ADMEt assays, amitraz and its metabolites were predicted to be compounds that are able to pass the blood–brain barrier (BBB) and induce toxicity in the central and peripheral nervous systems. The main target class predicted for amitraz was the family of A G protein-coupled receptors that comprises responses to hormones and neurotransmitters. This affects, among other things, reproduction, development, locomotion, and feeding. Furthermore, amitraz and its metabolites were predicted as active compounds interacting with diverse receptors of the Tox21-nuclear receptor signaling and stress response pathways.

## 1. Introduction

Bees are exposed to a great variety of potentially toxic chemicals that are carried into the beehive by foraging honeybees. Nectar and pollen may contain environmental pollutants or systemic pesticides drawn from the soil, or they can be contaminated from topical pesticide applications or drift from such applications [1,2,3,4].

In the last decades, different insecticides have been applied within the beehive to control different parasites, such as Varroa mites, with amitraz being one of the most common pesticides used by the beekeepers to control them [5]. The *Varroa jacobsoni* (today called *Varroa destructor*) causes the most destructive disease in honeybees worldwide, inflicting much greater damage than other known parasitic diseases and higher economic costs than all other apicultural diseases. Thus, amitraz has been widely used to counteract Varroa mites due to the high efficacy shown against the parasite [6,7].

Pesticides used for beehive treatments could be incorporated into the honey and accumulate in other hive products. Certain pesticides are carcinogenic and others may cause dysfunctions of the nervous and reproductive systems. Hence, honeybee health and the impairment of the quality of honey caused by pesticides have attracted public concern and the attention of the scientific community [8]. In addition, these residues could be extremely harmful to customers, even when present in small doses in the final products. In this sense, it must be noted that the main difference between amitraz and other conventional insecticides (e.g., the organophosphates) is the relative importance of sublethal doses and behavioral effects derived, as compared to direct lethality.

Amitraz (N,N’-[(methylimino)dimethylidyne]di-2,4-xylidine) is a formamidine pesticide with a broad-spectrum insecticide and acaricide action. In particular, amitraz is widely used in agriculture and livestock as a treatment to control different parasites [3]. In plants, amitraz is rapidly degraded to N-(2,4-dimethylphenyl)-N0-methylformamidine (BTS 27271 or 2,4-DPMF) and N-(2,4-dimethylphenyl) formamide (BTS 27919 or 2,4-DMF) [9,10]. The hydrolysis pathway of amitraz shows that it is readily hydrolyzed (under acidic conditions) to two main compounds, as mentioned above (BTS 27271 and BTS 27919), which can be rapidly hydrolyzed to 2,4-dimethylaniline (2,4-DMA) under alkaline conditions.

Regarding its biological effects, amitraz acts as an agonist of the octopamine receptor. Octopamine is an important neurotransmitter and hormone in invertebrates, affecting a wide variety of physiological functions, including laying eggs, learning and memory, locomotion, flight, aggression, courtship, sleep, arousal, and feeding behavior. Despite its importance in invertebrates, it does not play an important role in mammals. However, the similarity between insect octopamine receptors and mammalian α_2_-adrenergic receptors (α_2_-adrenoceptors) indicates that adrenergic receptors can be a target for amitraz. In fact, both in vivo and in vitro studies have shown that amitraz acts as rather selective agonists for α_2_-adrenoceptors, inducing several of its neurotoxic effects mainly through the activation of these receptors [11,12]. 

Additionally, the chemical structure of the mammalian neurotransmitter norepinephrine and the insect neurotransmitter octopamine is closely related. Thus, as most structural components of the nervous system are similar or identical in insects and mammals, it can be stated that amitraz is capable of affecting the nervous system of mammals [12]. Diverse in vivo studies with amitraz and other formamidines have confirmed that α_2_-adrenoceptors are targeted by these compounds and by their active metabolites. In a mouse brain, the occupation of α_2_-adrenoceptors by formamidines significantly decreased the binding of clonidine, which is a known selective α_2_-adrenoceptor agonist, more specifically in the cerebral cortex, hippocampus, and midbrain areas [11]. Other studies have reported that the distribution of α_2_-adrenoceptors in the peripheral nervous system also allows for multiple peripheral actions of amitraz, causing, for example, a dose-dependent decrease in hepatic glutathione (GSH) in mice [12].

Upon stimulation with octopamine, a second messenger is released, causing neuroexcitation and leading to behavioral effects in honeybees. These effects include increased excitability, increased motor activity, and decreased feeding behavior. Thus, neurotoxic effects have been described to occur after amitraz exposure [12]. In addition, among neurotoxic effects, other side effects of amitraz on olfaction have been reported, affecting diverse aspects of the honeybee’s social life organization and nectar foraging behavior, all regulated by the olfaction mechanism [13].

Amitraz poisoning cases in humans and animals are still being described to date, which is a cause for concern for health authorities. The most common poisoning effects described are sedation or unconsciousness, bradycardia, hypotension, hyperglycemia, and respiratory depression. Activation of α_2_-adrenoceptors in the central nervous system by amitraz and/or its metabolites has been described as the main mechanism for the altered mental status (sedation and unconsciousness) and for other adverse effects, such as lowered blood pressure and bradycardia, due to the diminished peripheral sympathetic tone. On the other hand, hyperglycemia can be explained by the action of amitraz on α_2_-adrenoceptors in the pancreas, whose activation inhibits the release of insulin [12].

Different mechanisms of amitraz toxicity have been described as such: agonist of α-adrenergic receptors and inhibition of the histamine H_1_ receptor, prostaglandin synthetase, monoamine oxidase (MAO), adenylyl cyclase, activation of voltage-dependent calcium ion (Ca^2+^) channel, and reactive oxygen species (ROS) production and endocrine disruptor. Furthermore, some of these mechanisms have been described to be implicated in seizure induction, highlighting its neurotoxic potential. However, all these effects could not fully explain the action of amitraz, as nowadays, there is still a controversy concerning the mechanisms by which amitraz exerts neurotoxic effects such as the reduction of motor activity, aggressiveness, and seizure induction [11].

Data on amitraz toxicity associated to cancer, genotoxicity, oxidative stress, cell death, neurotoxicity, immunotoxicity, endocrine disruption, and other developmental toxicity have been documented in earlier studies conducted by the Environmental Protection Agency (EPA) and the Joint Meeting on Pesticide Residues (JMPR) [14]. This information suggests that the danger posed by this substance might be overstated. Additionally, there is a lack of knowledge on the dose–response relationship for several mechanisms and toxic effects documented for amitraz and its metabolites, the mode of action that causes several toxic effects, and the pharmacokinetics of amitraz on various species.

According to the OCDE, alternative methods must replace in vivo methods, as far as possible, to determine toxic effects of contaminants [15]. Among alternative methods, both in vitro and in silico strategies can be applied. On the one hand, in vitro toxicity testing models using cell cultures are extensively used for detecting pesticides’ adverse effects. They reveal important insights about their cytotoxicity and are useful for better understanding of the mechanisms of action [16]. On the other hand, in silico technology has been widely used in recent years to evaluate the relevant properties of pesticides [17]. Several guidance documents have been drafted to improve standardization, harmonization, and uptake of in silico methods by regulatory authorities including the EFSA [18] and the ECHA (European Chemicals Agency). These methods are usually used together with other toxicity tests, and include models based on experimental data, quantitative structure-activity relationship (QSAR), and scientific data. 

The predictive SwissADME, SwissTargetPrediction, and ProTox-II tools are considered as valid alternatives to experimental procedures [19]. They are robust, fast, and are easy to use. However, some drawbacks and limitations can be found when using these alternative methods. On the one hand, in silico predictions must be critically assessed and an expert review of the output is often necessary. On the other hand, a deep knowledge on how the computational models implemented in the available free tools have been developed is sometimes not possible and thus, it is not possible to know prior to the prediction if the molecules intended for prediction are in the applicability domain or whether similar compounds have been included in the training set used to build the computational model.

The objective of this investigation was to study the mechanisms of action related to the cytotoxic effect produced by the pesticide amitraz and its metabolites (2,4-DMF and 2,4-DMA) in human hepatocarcinoma HepG2 cells, and to evaluate possible target receptors by in silico strategies. This procedure may allow us to prioritize substances for further in-depth toxicological evaluation as well as to identify some mechanisms for further investigation, such as disease-associated pathways.

## 2. Materials and Methods

### 2.1. Reagents

The reagent grade chemicals and cell culture compounds used, namely Dulbecco’s Modified Eagle’s Medium (DMEM), penicillin, streptomycin, trypsin/EDTA solutions, Phosphate Buffer Saline (PBS), Newborn Calf Serum (NBCS), methylthiazoltetrazolium salt (MTT) dye, dimethyl sulfoxide (DMSO), NaOH, NaCl, HEPES, and CaCl_2_ were purchased from Sigma-Aldrich (St. Louis, MO, USA). Methanol (MeOH) was purchased from VWR International (LLC, Radnor, PA, USA). Deionized water (resistivity < 18 MΩ cm) was obtained using a Milli-Q water purification system (Millipore, Bedford, MA, USA).

**Figure 1:**
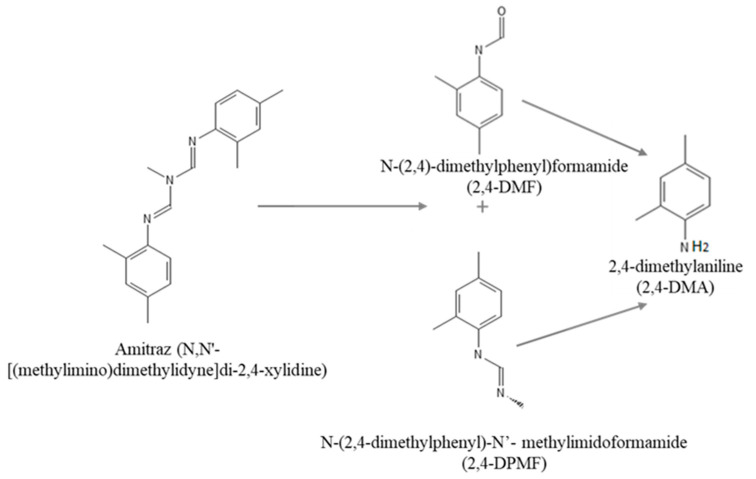
Hydrolysis pathway of amitraz (under acidic conditions) to its main compounds (DMF and DPMF), which can by rapidly hydrolyzed to 2,4-DMA under alkaline conditions.

The standards of the pesticide amitraz (MW 293.41 g/mol), 2,4-dimethylaniline (2,4-DMA; MW 121.18 g/mol), and n-(2,4-dymethylphenyl) formamide (2,4-DMF; MW 149.19 g/mol) were purchased from Sigma-Aldrich (St. Louis, MO, USA). Hydrolysis pathway of amitraz and chemical structures of amitraz and its modified forms are shown in Figure 1. Stock solutions of amitraz and 2,4-DMA were prepared in methanol, and 2,4-DMF in DMSO, all of them at appropriate working concentrations and maintained in the darkness at −20 °C.

### 2.2. Cell Culture and Treatment

Human hepatocarcinoma (HepG2) (ATCC: HB-8065) cells were cultured in DMEM medium supplemented with 10% NBCS, 100 U/mL penicillin, and 100 mg/mL streptomycin. The incubation conditions were pH 7.4, 5% CO_2_ at 37 °C, and 95% air atmosphere at constant humidity. The medium was changed every 5 days. The final pesticide concentrations tested were achieved by adding each pesticide solution to the culture medium with a final MeOH or DMSO concentration ≤ 1% (*v*/*v*). Appropriate controls containing the same amounts of solvents were included in each experiment. Absence of mycoplasma was checked routinely using the MycoAlert^TM^ PLUS Mycoplasma Kit (Lonza, Rockland, ME, USA).

### 2.3. In Vitro Cytotoxicity

Cytotoxic effects were determined in HepG2 cells by the MTT and total protein content (PC) assays. They have been extensively used in in vitro toxicological studies for measuring cell proliferation and survival. The MTT assay determines the viability of cells by the reduction of yellow soluble tetrazolium salt (MTT), only in the metabolically active cells, via a mitochondrial-dependent reaction to an insoluble purple formazan crystal. The MTT viability assay was performed according to Ruiz et al. (2006) [20]. To begin, 2 × 10^4^ cell/well were plated in 96-well tissue culture plates. After cells reached 80% confluence, the culture medium was replaced with fresh medium containing serial dilutions of each pesticide (amitraz from 46.88 to 625 µM, 2,4-DMF from 93.75 to 1500 µM, and 2,4-DMA from 93.75 to 1500 µM). The pesticides were exposed at 24, 48, and 72 h. During the exposure time, neither the medium nor the pesticides was replenished. After the exposure time, the medium was removed and 200 μL of fresh medium was added to each well. Then we added 50 μL/well of MTT and the plates were returned to the incubator in the darkness. After 3 h of incubation, the MTT solution was removed and 200 μL of DMSO was added, followed by 25 μL Sorensen’s glycine buffer. Plates were gently shaken for 5 min to achieve the complete dissolution. The absorbance was measured at 540 nm using an automatic ELISA plate reader (MultiSkanEX, Thermo Scientific, Waltham, MA, USA). 

The PC method is based on the increase of absorbance of Coomassie Brilliant Blue dye when binding to proteins. The assay was performed in the same 96-well plates where the MTT test was carried out. First, the plates were washed with PBS and each well received 200 μL of NaOH 0.1 N to dissolve the proteins. After 2 h of incubation, 170 μL of NaOH was removed from each well and 180 μL of diluted 22% Coomassie Brilliant Blue was added. The plates remained for 30 min at room temperature and the absorbance was measured at 620 nm in an automatic ELISA plate reader (MultiSkanEX, Thermo Scientific, MA, USA). 

For MTT and PC assays, cell viability was expressed as a percentage relative to the control solvent (≤1% MeOH or DMSO). Three independent experiments were conducted for each exposure time and at least in quadruplicate per concentration. The mean inhibition concentration (IC_50_) values were calculated using SigmaPlot version 11 (Systat Software Inc., GmbH, Germany).

### 2.4. Statistical Analysis

The statistical analysis of the data was carried out using the SPSS version 24.0 statistical package (IBM Corp., Armonk, NY, USA). Data were expressed as mean ± standard error of the mean (SEM) of the different independent experiments. The statistical analysis of the results was performed using the Student’s *t*-test for paired samples. Differences between groups were analyzed using one-way analysis of variance (ANOVA) followed by the Tukey HDS post hoc test for multiple comparisons. Statistical significance was considered for *p* ≤ 0.05.

### 2.5. In Silico ADME Profile Prediction for Toxicokinetics of Amitraz and Its Modified Forms

Predictive models to determine absorption, distribution, metabolism, excretion, and toxicity (ADMET) constitute valid alternatives to experimental assays, because of their robustness, speed, ease of interpretation, and ability to efficiently translate to risk assessment through molecular design.

The ADMET profiles of the three molecules amitraz, 2,4-DMF, and 2,4-DMA were predicted using SwissADME tools (http://www.swissadme.ch/, accessed on 2 November 2022) and admetSAR (http://lmmd.ecust.edu.cn/admetsar2/, accessed on 2 November 2022). To evaluate the absorption rate, necessary for oral administration, the following parameters were analyzed: the number of free rotatable bonds (n-ROTB) and Lipinski’s “rule of five” for the lead compounds. Lipinski’s descriptors evaluate the molecular properties for drug pharmacokinetics in the human body, especially for oral absorption. The rule states that the most “druglike” molecules present clogP ≤ 5, molecular weight (MW) ≤ 500, number of hydrogen bond acceptors (HBA) ≤ 10, and hydrogen bond donors (HBD) ≤ 5.33.

The main parameters for absorption, distribution, metabolism, and toxicity profile prediction of amitraz and its metabolites were taken into account according to the in silico ADME tool selected.

### 2.6. In Silico Tool “SwissTargetPrediction” for Target Prediction of Amitraz and Its Modified Forms

A predictive webserver tool “SwissTargetPrediction” (http://www.swisstargetprediction.ch/, accessed on 2 November 2022) has been used to predict possible targets of amitraz and its two metabolites, 2,4-DMF and 2,4-DMA. “SwissTargetPrediction” provides an intuitive interface to predict small molecule protein targets. This tool is based on the statement that similar bioactive molecules are more likely to share similar targets. In “SwissTargetPrediction”, both 2D and 3D similarity values are computed against a set of known ligands.

The tool prediction system evaluates only the interaction of a particular structure (in this case amitraz, 2,4-DMF, and 2,4-DMA) with molecular targets, giving as an output the target name, its identifiers in the UniProt and ChEMBL databases, the probability of assignment of the compound to the particular target, and the number of similar compounds having the same target.

Query molecules (amitraz, 2,4-DMF, and 2,4-DMA) have been analyzed by inputting the SMILES directly in the web interface and then selecting the organism in which predictions should be made (human, mouse, and rat). In this case all predictions have been calculated for humans and the SMILES for all three molecules analyzed have been taken from PubChem (https://pubchem.ncbi.nlm.nih.gov/, accessed on 2 November 2022). The SMILES is first checked to ensure that it corresponds to a valid chemical structure. If true, the similarity between the query molecule and all ligands in the database is computed and the score of each target is derived from the combined 2D and 3D similarity values with the most similar ligands. Targets are ranked according to their score with respect to the query molecule.

### 2.7. In Silico ProTox-II Tool for Toxicological Pathways Prediction of Amitraz and Its Modified Forms

ProTox-II (https://tox-new.charite.de/protox_II/, accessed on 2 November 2022) provides a freely available webserver for in silico toxicity prediction. This tool prediction method is based in molecular similarity, pharmacophores, fragment propensities, and machine-learning models for the prediction of various toxicity endpoints. In this study, ProTox-II has been used to evaluate the adverse outcome pathways (Tox21) and possible toxicity targets of amitraz ant its metabolites, 2,4-DMF and 2,4-DMA. Two types of target pathway-based models are implemented in ProTox-II: nuclear receptor signaling pathways (7 pathway assays: aryl hydrocarbon receptor (AhR), androgen receptor (AR), androgen receptor ligand binding domain (AR-LBD), aromatase (Aro), estrogen receptor alpha (ERα), estrogen receptor ligand binding domain (ER-LBD), and peroxisome proliferator activated receptor gamma (PPAR-gamma)) and stress response pathways (5 pathway assays: nuclear factor (erythroid-derived 2)-like 2/antioxidant responsive element (Nrf2/ARE), heat shock factor response element (HSE), phosphoprotein (tumor suppressor) p53, mitochondrial membrane potential (MMP), and ATPase family AAA domain containing protein 5 (ATAD5)). To perform predictions, the SMILES (consulted in PubChem) of each compound has been used as input in the webserver to calculate all parameters.

All the models implemented in the webserver tool have been validated on independent external sets and have shown strong performance. Additionally, all the predictive models for toxicology pathways have been implemented as toxicology in the 21st Century (Tox21), which is a federal collaboration among the United States Environmental Protection Agency (EPA) and the National Institute of Health (NIH), including the National Centre for Advancing Translational Sciences, the National Toxicology Program at the National Institute of Environmental Health Sciences, and the Food and Drug Administration [21].

## 3. Results

### 3.1. Cytotoxic Effect of Amitraz and Its Metabolites

**Figure 2:**
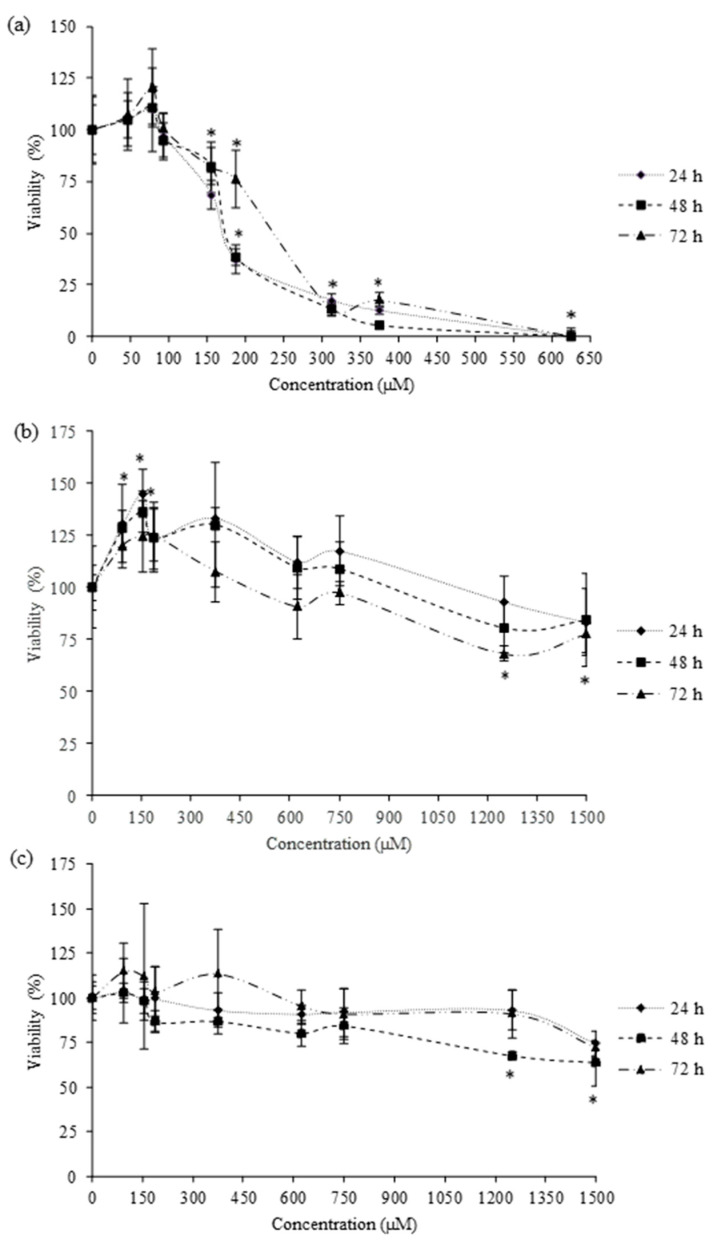
Cell viability of (**a**) amitraz, (**b**) 2,4-DMF, and (**c**) 2,4-DMA on HepG2 cells by MTT assay after 24, 48, and 72 h of exposure. Results are expressed as mean ± SEM (n = 3). (*) *p* ≤ 0.05 indicates significantly differences from control.

The cytotoxic effect of amitraz and its metabolites was evaluated by the MTT assay and PC assays at 24, 48, and 72 h of exposure. Figure 2 shows the dose–response curve for amitraz, 2,4-DMF, and 2,4-DMA after 24, 48, and 72 h. 

As shown in Figure 2a, amitraz produced similar decreases in cell viability at the three times of exposure when tested by MTT assay. Amitraz significantly (*p ≤* 0.05) decreased cell viability from 31% to 100% at concentrations ranging from 187.5 to 625 μM. As shown in Figure 2b,c, no significant reduction in cell viability was produced by 2,4-DMF and 2,4-DMA in HepG2 cells after 24, 48, and 72 h of exposure. The 2,4-DMF significantly (*p ≤* 0.05) decreased cell viability (from 22% to 32%) at the higher concentrations tested (1250 and 1500 µM) after 72 h of exposure. The 2,4-DMA decreased from 26% to 36% HepG2 cell viability after 24, 48, and 72 h. 

**Figure 3:**
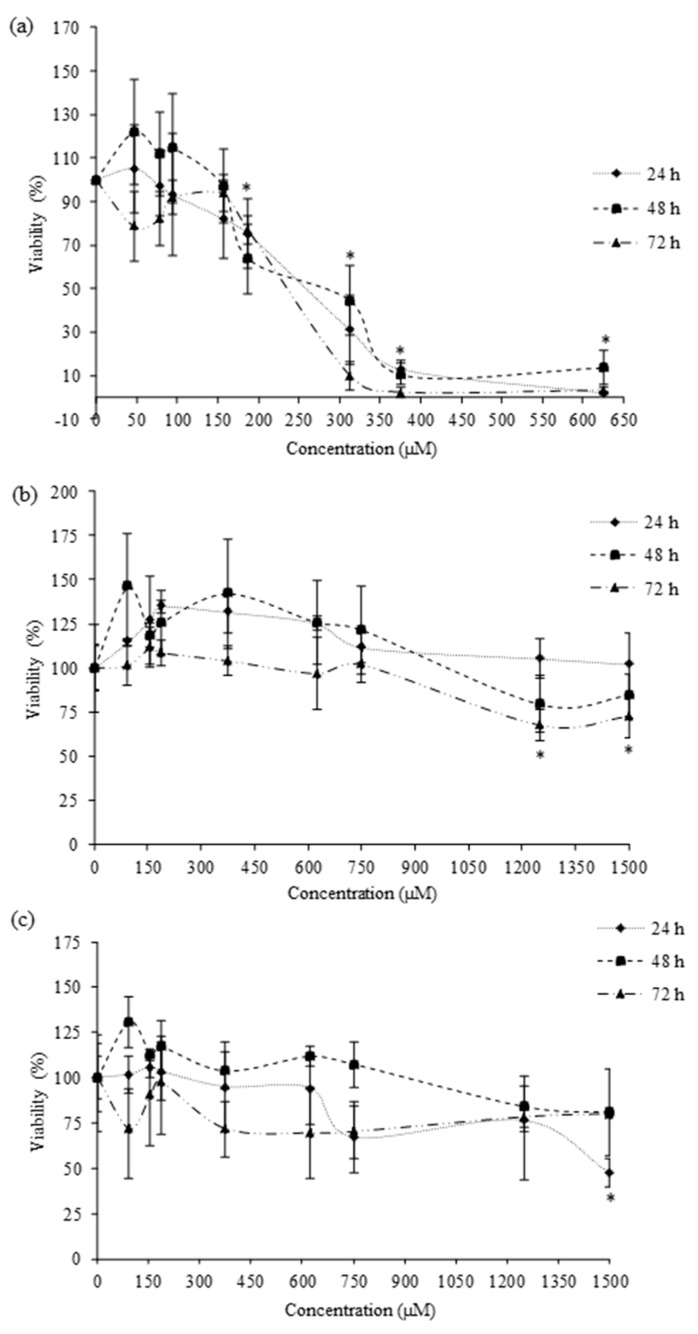
Cell viability of (**a**) amitraz, (**b**) 2,4-DMF, and (**c**) 2,4-DMA on HepG2 cells by total protein content assay after 24, 48, and 72 h of exposure. Results are expressed as mean ± SEM (n = 3). (*) *p* ≤ 0.05 indicates significantly differences from control.

Similar results to those obtained by MTT were obtained by PC assay for amitraz, 2,4-DMF, and 2,4-DMA (Figure 3). As shown in Figure 3a, amitraz produced similar decreases in cell viability at the three times of exposure when tested by PC assay. Significant (*p ≤* 0.05) reduction of cell viability (24–100%) was induced by amitraz from 187.5 μM. However, no significant reduction in cell viability was produced by 2,4-DMF and 2,4-DMA in HepG2 cells after 24, 48, and 72 h of exposure by PC assay (Figure 3b,c). The 2,4-DMF significantly (*p ≤* 0.05) decreased cell viability, from 27% to 32% at 1250 and 1500 µM after 72 h of exposure, respectively. The 2,4-DMA significantly (*p ≤* 0.05) decreased cell viability to 53%, at 1500 µM after 24 h of exposure.

Table 1 shows the IC_50_ (µM ± SEM, n = 3) values of amitraz and its metabolites in HepG2 cells after 24, 48, and 72 h of exposure. The IC_50_ values were obtained in HepG2 cells exposed to amitraz at the three times of exposure, whereas 2,4-DMF and 2,4-DMA, being less cytotoxic, did not show any IC_50_ value at the range of concentrations tested at all times of exposure. 

### 3.2. In Silico ADMET Profile Prediction for Amitraz and Its Metabolites

Pesticide-likeness qualitatively assesses the probability of a molecule of becoming an oral pesticide with respect to bioavailability. The SwissADME gives access to 5 different rule-based filters. The Lipinski filter is the pioneer of the “rule of five”. Lipinski’s molecular descriptors and the predicted data of some ADMET properties of amitraz, 2,4-DMF, and 2,4-DMA are summarized in Table 2. 

The Lipinski filter was selected in this study. Both amitraz and its metabolites showed acceptable n-ROTB values (≤10) and complied with all conditions of Lipinski’s rule, except for the amitraz that has clogP slightly higher than 5.

Inter- and intraspecies differences in toxicokinetics (absorption, distribution, metabolism, and excretion) can influence the concentration of a chemical in the brain and thereby influence the risk of a chemical to exert neurotoxicity. Thus, the study of important toxicokinetic parameters has been calculated. Gastrointestinal absorption (HIA), oral bioavailability (HOB), and brain penetration (BBB) are positive for all of the molecules. 

All pesticides showed moderate to high gastrointestinal absorption (Caco-2 cell permeability and HIA, Table 2). The results, after an overview of the BOILED-Egg construction, demonstrated that the three molecules have the highest probability of being absorbed by the gastrointestinal tract and they are likely to permeate into the brain (Table 2). In the case of metabolism, various cytochrome P450 isoenzymes were evaluated, showing different patterns for all the compounds. In terms of toxicity, amitraz showed toxicity in hepatic cells; the metabolite 2,4-DMA showed toxicity in relation to the AMES test; and none of the molecules showed carcinogenic effects. All of them showed toxicity in relation to aquatic fish and crustaceans. No honeybee cytotoxicity was observed.

### 3.3. In Silico Tool “SwissTargetPrediction” for Target Prediction of Amitraz and Its Modified Forms

**Figure 4:**
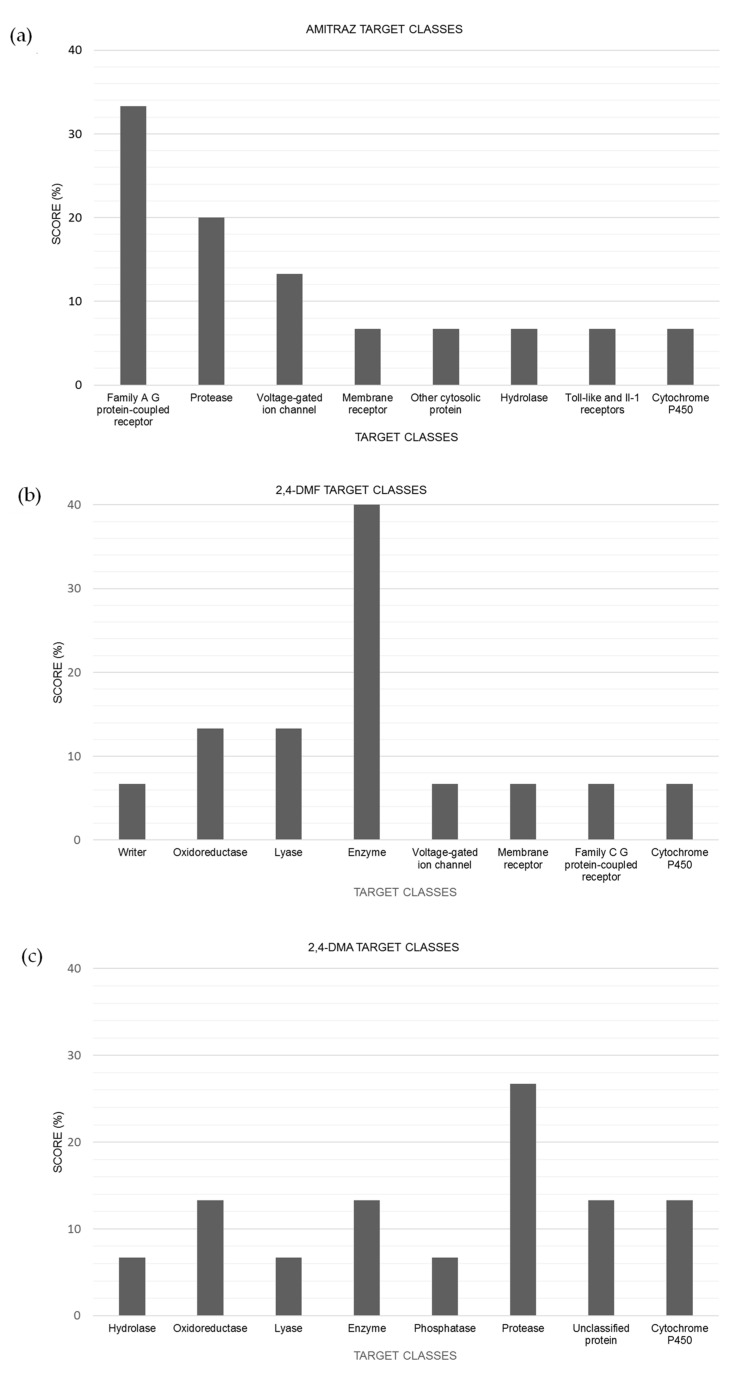
Target classes predicted for (**a**) amitraz, (**b**) 2,4-DMF, and (**c**) 2,4-DMA in “SwissTargetPrediction”. The graphic shows the percentage of the predicted target classes for analyzed compounds, whose percentages have been calculated using the top 15 predicted targets.

“SwissTargetPrediction” software has been used to predict possible targets of amitraz and their two main metabolites (2,4-DMF and 2,4-DMA). As it can be observed in Figure 4a, the main target class for amitraz is the family of A G protein-coupled receptors, showing a 35% of prevalence among the top 15 target classes. More concretely, the probability of amitraz to have this protein as a target is 100%. It is known that the α2-adrenoceptors belongs to the G protein-coupled receptor (GPCR) superfamily and thus this class is likely the main target receptor for amitraz. Other predicted targets included in the top 15 which belong to A G protein-coupled receptor were kappa opioid receptor, serotonin 1d (5-HT1d) receptor, C-C chemokine receptor type 3, and trace amine-associated receptor.

Regarding 2,4-DMF predicted targets, the main target class (40%) in the top 15 was the enzyme class (Figure 4b), including, as the most probable enzymes: myeloperoxidase, thymidine phosphorylase, arachidonate 12-lipoxygenase, indoleamine 2,3-dioxygenase, poly [ADP-ribose] polymerase-1, and leukocyte common antigen.

For 2,4-DMA predicted targets, the most abundant class (27%) corresponded to the protease group, the most probable proteases acting as targets being: trypsin I, urokinase-type plasminogen activator, plasminogen, and kallikrein 1. 

### 3.4. In Silico ProTox-II Tool for Toxicological Pathways Prediction of Amitraz and Its Modified Forms

ProTox-II has been used for the prediction of Tox21 nuclear receptor signaling and stress response pathways of amitraz and its metabolites. In relation to the nuclear receptor signaling endpoint, amitraz has been predicted to be an active compound to interact with the AhR, which is a protein known for its role in mediating toxicity (Table 3). The 2,4-DMF did not show any activity to interact with the AhR. However, 2,4-DMA was predicted to be an active molecule able to interact with both the AhR and the ERα (Table 3).

Regarding the in silico prediction of Tox21 stress response pathways, as it can be observed in Table 4, amitraz was shown to be active for the MMP. This is an essential component in the process of energy storage during oxidative phosphorylation. Amitraz metabolites 2,4-DMF and 2,4-DMA, did not show any activity in the predicted stress response pathways (Table 4).

## 4. Discussion

In the present study amitraz toxicity together with its metabolites’ toxicity have been investigated. It is well known that, on acute exposure, amitraz can induce serious dysfunction in animals and humans [22,23]. The HepG2 cells have been selected as the experimental system in vitro to detect toxicity induced by amitraz, 2,4-DMF, and 2,4-DMA, since the liver is the initial organ where the toxins are being metabolized. The HepG2 cells retain the activities of various phase I and II enzymes and have a high number of mitochondria organelles [24]. However, HepG2 cells have been shown to express low cytochrome P450 activities and thus low biotransformation activity in comparison with the human liver [25]. The HepG2 cells were also shown to be slightly more sensitive to cytotoxic compounds compared to other commonly used cell lines, such as HeLa or Caco-2 cells. 

Amitraz and its metabolites’ cytotoxicity in HepG2 cells has been assessed. Numerous in vitro cytotoxicity assays, with different endpoints, are available for cell culture. Evaluating the results obtained in this study by MTT and PC assays, we can conclude that both of them are sensitive and comparable to evaluate amitraz and its metabolites’ cytotoxicity (Table 1). However, amitraz and its metabolites have been scarce tested to determine their cytotoxicity in cell cultures, and none of those studies report IC_50_ values. The cytotoxic effect of amitraz (from 60 to 120 µM) on primary hippocampal cells was evaluated using the MTT assay for 24 h. Amitraz decreased the cell viability in a concentration-dependent manner up to 100 µM [26]. Similarly, del Pino et al., observed a decrease in metabolic activity of primary hippocampal cells exposed to 100 µM amitraz by MTT assay for 24 h [11]. The cytotoxicity of the amitraz (from 0.119 to 119 µM) in human lymphoblastoid (WIL2NS) cell line was also evidenced after 24, 48, and 72 h by [27]. They observed a significant decrease in cell viability for all concentrations tested, except for the lowest concentration assayed (0.119 µM). 

As observed, amitraz and its metabolites could be dangerous and damage cells or even a whole organism. Therefore, to know their potential risk, it is important to know their absorption and fate in organisms and their ability to affect the brain. In this sense, the next objective was to predict the toxicity of amitraz, 2,4-DMF, and 2,4-DMA with an in silico predictive behavior method, the SwissADMEt method. According to Lipinski’s “rule of five”, amitraz and its metabolites exhibit good absorption or permeation after oral exposure, and they have shown the ability to pass the blood–brain barrier (BBB). Indeed, these results indicate that amitraz can induce toxicity in the central nervous system (CNS), also inducing toxic effects in the peripheral nervous system as they are able to cross the BBB.

As evidenced, amitraz, 2,4-DMA, and 2,4-DMF are soluble in octanol rather than in water (high logP value). The liposolubility of these pesticides favors bioavailability (HOB) manifested by their high penetration through the BBB (Table 2). The 2,4-DMA is substrate of CYP450 2D6 enzyme, and amitraz is an inhibitor of the CYP450 2C19 enzyme, as both of them are involved in the Phase I oxidative metabolism. These findings are of great relevance, because there may be competition between substances that are metabolized by the same enzymes. On the other hand, with respect to amitraz, 2,4-DMA, and 2,4-DMF transporters, the admetSAR tool indicates that transporter inhibition has been observed (BSEP, OATP1b1 and OATP1b3; Table 2). 

With respect to genotoxic toxicity data, carcinogenicity was negative for the three pesticides and the micronucleus test was positive for all of them. However, with respect to the Ames assay, 2,4- DMA was the only compound that showed a mutagenic response. With regard to ecotoxicity tests, the results show toxicity in fish aquatic and crustacean aquatic tests. However, as expected none of the tested pesticides induced honeybee toxicity by the predictive toxicity method selected in this study (Table 2). Therefore, all these findings from predictive toxicity could contribute to a better knowledge about the potential risk of toxicity from these pesticides to human and animal health. 

Regarding other results provided by diverse in silico tools, amitraz and its metabolites have shown their ability to act as active compounds that can interact with diverse receptors. This approach is based on the fact that a chemical compound can activate or inhibit a receptor or an enzyme when it interacts with them, resulting in a perturbation in diverse biological pathways, thereby disrupting the cellular process and causing cell death.

The main target class predicted by in silico tools for amitraz was the family of A G protein-coupled receptors (Figure 4a). G protein coupled receptors (GPCRs) are integral membrane proteins comprising a large family of membrane-bound receptors whose main function is to convert extracellular signals into intracellular responses, thus regulating different cell signaling pathways. The most common responses comprise responses to hormones, neurotransmitters, as well as responses to vision, olfaction, and taste signals. In insects, they regulate major biological processes such as reproduction, development, locomotion, and feeding [28]. Thus, the interaction of amitraz with these receptors can explain some of the effects produced by pesticide exposure.

Moreover, it has been demonstrated that the G protein exerts an inhibitory effect when coupled to α_2_-adrenoceptors, thus, resulting in the subsequent inhibition of adenylyl cyclase, phospholipase C (PLC), influx of intracellular calcium (Ca^+^), and increased efflux of potassium (K^+^) ions [12,29]. In the literature, amitraz and its metabolites have been reported to be potent inhibitors of acetylcholinesterase [30]; indeed, they have been predicted to be a candidate to interact with some enzymes involved in different pathways (Figure 4b,c). 

Furthermore, amitraz and its metabolites have been predicted to interact with some key receptors of the Tox21 nuclear receptor signaling and the stress response pathways. Regarding the nuclear receptor signaling pathways, both amitraz and 2,4-DMA were predicted as compounds able to interact with the AhR, which is a protein known for its role in mediating toxicity. It has been described that the activation of the AhR can induce immunotoxicity, including thymic involution. In this sense, recent data suggest that AhR plays an important role in T-cell and T-helper 17 differentiation [31]. In addition, it must be highlighted that amitraz has been predicted by SwissADME tools as an active compound that will be able to bind to the thyroid receptor (Table 2). 

Several reports have described the ability of amitraz to disrupt hormones by different mechanisms, mainly through the activation of the α_2_-adrenergic receptors [11]. In this study, in silico tools could predict a possible interaction between amitraz and its metabolites with hormone receptors. Thus, 2,4-DMA has been predicted as an active compound to interact with the ER by ProTox-II, while amitraz has been predicted as an active compound to interact with ER by the SwissADME tool. The ER is located at the peri-membrane, mitochondria, and the nucleus of cells that are dependent on estrogen target tissues. This receptor has shown to be relevant in the induction of toxicity exerted by amitraz exposure, as it has been reported that the amitraz effects produced on different neurotransmitters can be partially blocked by antagonizing ER activities [32]. Other studies demonstrated that the administration of amitraz antagonized estradiol binding to the ER due to the replacement of the estradiol and the subsequent binding of amitraz to the receptor [33,34].

Finally, regarding the in silico prediction of Tox21 stress response pathways, amitraz was shown to be active for the MMP endpoint. This fact could indicate that amitraz is able to alter the process of energy storage during the oxidative phosphorylation. However, studies in this field are still scarce and further investigations are necessary to elucidate this mechanism. In addition, some limitations can be found in this study regarding the toxicity targets studied. The toxicity targets evaluated were those implemented in the free tool ProTox-II, and although important information was obtained, it would be interesting to evaluate other possible targets for amitraz and its metabolites, especially regarding their neurotoxic potential. 

In comparison to in vivo techniques, which are performed in whole organisms, alternative approaches, and more concretely in silico modelling, offer more practical and economical experiments. Furthermore, computational methods limit the use of animal models in research. Thus, in silico web tools have been used to predict different endpoints of amitraz and its metabolites, obtaining valuable information that could be useful in understanding the mechanism of action and the possible targets of amitraz and its metabolites, thus prioritizing and guiding future trials. In these tools, different QSAR models developed for each endpoint have been implemented. However, for QSAR model development, data compilation and data curation are essential. The predictive ability of the models depends heavily on the compounds used in the training set used to develop the prediction model. To this extent, the ability to assess the confidence in the predicted value is crucial for the correct interpretation and application of QSAR models. In fact, it must be understood that any resulting QSAR model is only as statistically valid as the data that led to its development. Due to the limitation in existing experimental data for some compounds, this fact can often be a serious drawback for efficient QSAR modelling. Moreover, as information on how QSAR models implemented in the web tools have been developed, is not possible to know beforehand if the molecules intended for prediction are in the applicability domain or whether these compounds or other similar ones, in terms of structure-activity, have been included in the training or validation sets used to build and validate the computational model.

Regarding in vitro cytotoxicity by MTT assay, this approach has extensive utility as a cell metabolic activity assay, showing some advantages including ease and rapidity of performance, reproducibility of the results, and observed clinical correlation between in vitro and in vivo testing. Due to its positive charge and lipophilic nature, the MTT reagent may pass through the mitochondrial inner membrane and cell membrane of live cells. Therefore, the MTT assay is one of the methods most frequently used to study cell toxicity. However, for some cell types, cytometry is a useful technique because the rate of formazan extrusion is modest enough to prevent bias in intracellular observations. Single-cell cytometry is often an effective method for determining the heterogeneity of a cell population. Because HepG2 cells are homogeneous, the MTT has been demonstrated to be a useful and acceptable test in this investigation to assess the cytotoxicity of amitraz and its metabolites.

Moreover, most of the drawbacks of the MTT technique have been taken into account in this study. Among the most common limitations of an MTT assay include the seeding cell number or the concentration of MTT reagent added to the cells, because reduction of the dye depends primarily on cell metabolism; and sometimes this is reflective of cell viability. The variability of the number of cells in the wells can be a drawback in the assay. For this reason, in the present study, this fact was compensated by the protein determination in the total protein assay. Other common problems that can occur include a decrease in the concentration of D-glucose, NADH, or NADPH in the culture medium which may be accompanied by a decrease in MTT-formazan production. To counteract this fact, the culture media have been tested previously and the MTT technique has been validated in previous studies carried out in our research group, achieving a homogeneity of the solubilized formazan and other tetrazolium-based compounds in the MTT assay. It has been also reported that the conversion to formazan crystals in an MTT assay depends on metabolic rate and number of mitochondria, which can result in interferences. In addition, in silico prediction of Tox21 stress response pathways performed in this study showed that amitraz can be considered an active compound for the MMP (mitochondrial membrane potential) endpoint, thus interacting with the above-mentioned effect. Thus, additional studies on the interaction between amitraz and its metabolites with different cellular components, especially mitochondria, are recommended.

## 5. Conclusions

In summary, this study demonstrated that the pesticide amitraz was more cytotoxic than 2,4-DMF and 2,4-DMA. According to the in silico ADMEt profile prediction, amitraz and its metabolites were positive for gastrointestinal absorption and brain penetration, and all of them could produce DNA damage by micronucleus generation. They could affect the aquatic environment, but they did not produce honeybee toxicity. 

Amitraz and its main metabolites, 2,4-DMF and 2,4-DMA, are known potent neurotoxicants. In honeybees they can lead to behavioral effects and neurotoxicity after the stimulation of octopamine receptors. The main neurotoxic effects include increased excitability, increased motor activity, and decreased feeding behavior. In humans, diverse poisoning cases pointed out the activation of α_2_-adrenoceptors in the central nervous system by amitraz and/or its metabolites as the main mechanism for the demonstrated neurotoxic effects. This can be explained by the similarity between octopamine receptors in honeybees and α_2_-adrenoceptors in humans and between the neurotransmitters octopamine and norepinephrine in insects and mammals, respectively. These structural similarities can be explored by in silico strategies, relating the chemical structure of a compound with a known biological activity. In this sense, amitraz and its metabolites have shown to be able to act as active compounds that can interact with diverse receptors of the Tox21 nuclear receptor signaling and the stress response pathways. The main predicted target of amitraz was the family of A G protein-coupled receptors, which has been described to exert an inhibitory effect when coupled to α_2_-adrenoceptors in humans. Thus, due to the similarities mentioned above, in honeybees, amitraz could interact with octopamine receptors, explaining some of the effects produced by exposure to amitraz.

## Figures and Tables

**Table 1:** The medium inhibitory concentration (IC_50_) of amitraz, 2,4-DMF, and 2,4-DMA in HepG2 cells determined after 24, 48, and 72 h of incubation by the MTT and PC assays. Results are mean ± SEM (n = 3). The IC_50_ values were calculated using SigmaPlot version 11 (Systat Software Inc., GmbH, Erkrath, Germany).

Assay	IC_50_ (µM) ± SEM								
	Amitraz			2,4-DMF			2,4-DMA		
	24 h	48 h	72 h	24 h	48 h	72 h	24 h	48 h	72 h
MTT	196.5 ± 10.1	175.8 ± 5.0	157.1 ± 10.1	>1500	>1500	>1500	>1500	>1500	>1500
PC	260.0 ± 13.3	212.8 ± 37.5	174.6 ± 2.9	>1500	>1500	>1500	>1500	>1500	>1500

**Table 2:** The ADME and toxicity profile prediction for amitraz, 2,4-DMA, and 2,4-DMF.

Parameters	Amitraz	2,4-DMF	2,4-DMA
Lipinski molecular descriptors	Yes; 1 violation	Yes; 0 violation	Yes; 0 violation
HBA (≤10)	2	1	0
HBD (≤5)	0	1	1
clogP (≤5)	5.50	1.47	1.68
MW (≤500 g/mol)	293.41	149.19	121.18
n-ROTB (≤10)	4	2	0
BOILED-Egg descriptors	Yes; 0 violation	Yes; 0 violation	Yes; 0 violation
Lipophilicity: Log Po/w (WLOG)	4.87	1.68	1.89
Water solubility (Log S(ESOL))	−5.26	−1.96	−2.14
TPSA (Å2)	27.96	29.10	26.02
Absorption			
HIA	(+)	(+)	(+)
HOB	(+)	(+)	(+)
Caco-2 permeability	(+)	(+)	(+)
Distribution			
P-gp substrate	(-)	(-)	(-)
BBB	(+)	(+)	(+)
Subcellular localization	Mitochondria	Mitochondria	Lysosomes
Metabolism			
CYP450 2C9 substrate	(−)	(−)	(−)
CYP450 2D6 substrate	(−)	(−)	(+)
CYP450 3A4 substrate	(−)	(−)	(−)
CYP450 1A2 inhibitor	(−)	(−)	(−)
CYP450 2C9 inhibitor	(−)	(−)	(−)
CYP450 2C19 inhibitor	(+)	(−)	(−)
CYP450 2D6 inhibitor	(−)	(−)	(−)
CYP450 3A4 inhibitor	(−)	(−)	(−)
BSEP inhibitor	(+)	(−)	(−)
OATP1b1 inhibitor	(+)	(+)	(+)
OATP1b3 inhibitor	(+)	(+)	(+)
Toxicity			
Hepatotoxicity	(+)	(−)	(−)
Honeybee toxicity	(−)	(−)	(−)
Fish aquatic toxicity	(+)	(+)	(+)
Crustacean aquatic toxicity	(+)	(+)	(+)
Acute oral toxicity (category *)	II	III	III
Estrogen receptor binding	(+)	(−)	(−)
Thyroid receptor binding	(+)	(−)	(−)
Glucocorticoid receptor binding	(−)	(−)	(−)
AMES mutagenesis	(−)	(−)	(+)
Carcinogenesis (binary)	(−)	(−)	(−)
Micronucleus assay	(+)	(+)	(+)

* BBB: blood-brain barrier; BBB (+): good penetrator of the BBB; BBB (−): poor penetrator of the BBB; BSEP: bile salt export pump pharmacokinetic transporter; Caco-2 (+): good permeability in an in vitro cellular permeability assay with cells by Caco-2 cells. clogP: logarithm of compound partition coefficient between n-octanol and water; HBA: number of hydrogen bond acceptors; HBD: number of hydrogen bond donors; HIA: human gastrointestinal absorption; HOB: human oral bioavailability; MW: molecular weight; n-ROTB: number of rotatable bounds; OATP1B1: organic anion transporting polypeptide 1B1 pharmacokinetic transporter; OATP1B3: organic anion transporting polypeptide 1B3 pharmacokinetic transporter; PPB: plasma protein binding; P-gp: P-glycoprotein; PK: pharmacokinetic transporter; P_o/w_: n-octanol/water partition coefficient; TPSA: polar surface area; II: Category II for pesticide corresponding to DL_50_ oral from 50 to 500 mg/Kg; III: Category III for pesticide corresponding to DL_50_ oral from 500 to 5000 mg/Kg. * The EPA established four toxicity categories for acute hazards of pesticide products, with category I being the highest toxicity category.

**Table 3:** Prediction of Tox21-nuclear receptor signaling pathways of amitraz and its metabolites.

Nuclear Receptor Signaling Pathway	Amitraz	2,4-DMF	2,4-DMA
AhR	Active (99)	Inactive (94)	Active (78)
AR	Inactive (98)	Inactive (100)	Inactive (100)
AR-LBD	Inactive (98)	Inactive (100)	Inactive (100)
Aro	Inactive (87)	Inactive (100)	Inactive (100)
ER	Inactive (92)	Inactive (95)	Active (85)
ER-LBD	Inactive (98)	Inactive (100)	Inactive (100)
PPAR-Gamma	Inactive (98)	Inactive (100)	Inactive (100)

AhR: aryl hydrocarbon receptor; AR: androgen receptor; AR-LBD: androgen receptor ligand binding domain; Aro: aromatase; ER: estrogen receptor; ER-LBD: estrogen receptor ligand binding domain; PPAR-Gamma: peroxisome proliferator activated receptor gamma.

**Table 4:** Prediction of Tox21- Stress response pathways of amitraz and its metabolites.

Stress Response Pathway	Amitraz	2,4-DMF	2,4-DMA
Nrf2/ARE	Inactive (95)	Inactive (100)	Inactive (100)
HSE	Inactive (95)	Inactive (100)	Inactive (100)
MMP	Active (100)	Inactive (95)	Inactive (99)
Phosphoprotein (tumor suppressor) p53	Inactive (95)	Inactive (100)	Inactive (100)
ATAD5	Inactive (93)	Inactive (100)	Inactive (100)

Nrf2/ARE: Nuclear factor (erythroid-derived 2)-like 2/antioxidant responsive element; HSE: heat shock factor response element; MMP: mitochondrial membrane potential; ATAD5: ATPase family AAA domain containing protein 5.

## Data Availability

Data is contained within the article.
